# An Insight into Knowledge, Perspective, and Practices of Indian Optometrists towards Childhood Myopia

**DOI:** 10.3390/vision8020022

**Published:** 2024-04-16

**Authors:** Archana Naik, Siddharth K. Karthikeyan, Jivitha Jyothi Ramesh, Shwetha Bhaskar, Chinnappa A. Ganapathi, Sayantan Biswas

**Affiliations:** 1 Department of Optometry, Netra Jyothi Institute of Allied Health Sciences, Udupi 576101, Karnataka, India; aarchu080@gmail.com (A.N.); siddharth.k@learner.manipal.edu (S.K.K.); shwethabhaskar06@gmail.com (S.B.); 2 Aloka Vision Programme, Carl Zeiss India Pvt. Ltd., Bangalore 560099, Karnataka, India; 3 Department of Optometry, Manipal College of Health Professions, Manipal Academy of Higher Education, Manipal 576104, Karnataka, India; jivitha.j@learner.manipal.edu; 4 Department of Paediatric Ophthalmology, Prasad Netralaya, Udupi 576101, Karnataka, India; dyanchi@gmail.com; 5 School of Optometry, College of Health and Life Sciences, Aston University, Birmingham B4 7ET, UK

**Keywords:** childhood myopia, myopia complications, myopia control, optometric practice, barriers, myopia management, clinical decision-making, survey

## Abstract

The current understanding of clinical approaches and barriers in managing childhood myopia among Indian optometrists is limited. This research underscores the necessity and relevance of evidence-based practice guidelines by exploring their knowledge, attitude, and practice towards childhood myopia. A self-administered internet-based 26-item survey was circulated online among practicing optometrists in India. The questions assessed the demographics, knowledge, self-reported clinical practice behavior, barriers, source of information guiding their management, and extent of adult caregiver engagement for childhood myopia. Of 393 responses, a significant proportion of respondents (32.6–92.4%) were unaware of the ocular complications associated with high myopia, with less than half (46.5%) routinely performing ocular biometry in clinical practice. Despite the growing awareness of emerging myopia management options, the uptake remains generally poor, with single-vision distance full-correction spectacles (70.3%) being the most common mode of vision correction. Barriers to adopting optimal myopia care are medicolegal concerns, absence of clinical practice guidelines, and inadequate consultation time. Own clinical experience and original research articles were the primary sources of information supporting clinical practice. Most (>70%) respondents considered involving the adult caregiver in their child’s clinical decision-making process. While practitioners’ awareness and activity of newer myopia management strategies are improving, there is plenty of scope for its enhancement. The importance of evidence-based practice guidelines and continuing education on myopia control might help practitioners enhance their clinical decision-making skills.

## 1. Introduction

Myopia or near-sightedness is a growing public health concern [1] and is predicted to affect half of the world’s population by 2050 [2]. The prevalence of myopia among urban Indian children is likewise expected to rise from 21.2% in 2019 to 48.1% by 2050 [3]. Myopia has significant social, educational, and economic consequences, reducing the quality of life [1], with high myopia (spherical equivalent ≤ −6.00 D) increasing the risk of sight-threatening complications and irreversible vision loss [4,5]. The earlier the age of myopia onset, the greater the risk of developing high myopia and related vision-threatening issues. Therefore, postponing the onset of myopia could delay or even prevent the development of pathological myopia [6].

There is an increasing interest in myopia management and control among practitioners worldwide [7]. Although refining environmental factors and lifestyle remains the optimal strategy for preventing or postponing the development of myopia, there are now various optical and pharmacological treatments accessible to decelerate the progression of the condition [8]. An array of childhood myopia control options exists, including glasses (single, bifocal, multifocal), contact lenses (soft, rigid, orthokeratology, peripheral defocus), pharmacological (topical atropine), and lifestyle changes (increased outdoor time) [8,9,10,11]. Emerging myopia control treatments have raised interest in the prevention and control of childhood myopia. Without clinical guidelines, effective strategies remain underused, with over 95% of practitioners relying on non-evidence-based single-vision glasses and advice on visual hygiene [12]. This highlights the urgent need for global clinical guidelines on myopia management [7,13,14,15]. There are several gaps in our current understanding of clinical practice pattern of childhood myopia. There are no published data relating to eye-care practitioners’ understanding of the consequences of myopia, diagnostic procedures, and treatment approaches for childhood myopia in India. Myopia management lacks any standard diagnostic protocol, natural history of myopia development, and risk of comorbidity for clinicians. There is also a need for insight into the knowledge base or evidence clinicians use to guide their clinical practice without regulatory approval for emerging myopia treatments. Finally, even less is known about the clinician and adult caregiver interaction concerning prescribing the most appropriate myopia management strategy.

Understanding the knowledge and practice patterns may suggest ways to enhance optometrists’ approaches to managing childhood myopia. This could involve targeted educational interventions, updated clinical guidelines, increased awareness of evidence-based practices, and improved caregiver communication strategies. The previous study conducted [16] on childhood myopia practice patterns in India is limited to inexperienced optometrists (~80% with <5 years of clinical experience) and only from a pocket in northern India (regional), not reflecting the diverse practice patterns throughout the country [17]. Given the increasing global prevalence of myopia and its long-term implications for eye health, it is crucial to understand Indian optometrists’ approaches to diagnosing and managing childhood myopia. By addressing knowledge gaps and enhancing clinical practices, we can improve the quality of care for young patients with myopia, leading to better outcomes and reduced long-term ocular risks.

Thus, this survey aims to explore and provide updated information on practicing optometrists’ knowledge, perspective, and practices in diagnosing and managing childhood myopia in India. Furthermore, it assesses the evidence guiding optometric practice and the involvement of adult caregivers in decisions regarding myopia in children.

## 2. Methods

An online survey using Google Forms (Google Inc., Menlo Park, CA, USA) was administered to practicing optometrists in India between 27 March 2020 and 27 September 2022. Ethics approval for the study was obtained from the Mangala Institutional Ethical Committee, and the study complied with the tenets of the Declaration of Helsinki.

### 2.1. Practitioner’s Survey

The questionnaire used in this study was adapted from a survey conducted among Australian optometrists [14]. Following a focus group discussion comprising four optometrists with experience of >5 years in clinical practice, the questionnaire was adopted to fit the scope and context of Indian optometric practice. The content validity was kept the same through a focused group discussion that resulted in the final version of the survey. We omitted the question about therapeutic endorsement, as it is not recognized in Indian optometry. The draft was piloted among five optometrists and five final-year optometry students to ensure the questions’ relevance, accuracy, clarity, and interpretation. The required time to complete the survey was approximately 7–10 min. The survey was distributed to optometrists working in India through state and national optometric associations via email, WhatsApp, Telegram, Facebook, and India Vision Institute’s E-Newsletter. Reminders to fill in the survey were sent via email and social media every two weeks during the study period. The survey consisted of a statement at the beginning that the participation is voluntary, and their submission of responses reflects their consent.

We included demographic questions (education and occupation) to filter out opticians and students. Only responses from optometrists practicing in India were considered—the survey aimed to understand their childhood myopia knowledge and practices. Duplicates were prevented with email address restrictions and questions on prior survey participation. Participation was voluntary, with no incentives. Responses were kept anonymous and treated confidentially.

#### Sample Size

The survey reached approximately 10,000 optometrists. To ensure a representative response with a margin of error of ±5% and a confidence level of 95%, a sample size of 370 responses was deemed necessary [18].

### 2.2. Questionnaire Design

This survey was a 26-item questionnaire which is described in detail elsewhere [14], mainly aimed to understand the practicing optometrists’:

- Understanding of the natural history and ocular complications associated with myopia.
- Clinical practice behavior related to the diagnosis and management of myopia in children aged 16 years or younger.
- Potential barriers to providing optimal myopia care.
- Source of evidence that clinicians use as a guide to their practice.
- Extent to which adult caregivers are involved during the decision-making process in treating childhood myopia.

Out of the 26 questions, there were 8× multiple choice questions, 6× matrix, 3× open-ended, 3× closed-ended, 3× dropdown, 1× Likert scale, 1× ranking, and 1× mixed type question (provided as Supplementary Material).

The definition of myopia was set at a spherical equivalent of −0.50 D or higher. Respondents were expected to complete the survey questions in the order provided by the authors and had no option to change the answer after submission. The Checklist for Reporting Results of Internet E-Surveys (CHERRIES) guidelines was used to prepare the survey (Table 1) [19].

### 2.3. Data Analysis

The online responses from Google Forms were imported into a Microsoft Office Excel 2016 spreadsheet for analysis. Statistical analyses were performed using Statistical Package for Social Sciences [IBM SPSS Statistics for Windows, Version 28.0, IBM Corp, Armonk, NY, USA], and graphs were plotted in GraphPad Prism version 6.04 (GraphPad Software Inc., La Jolla, CA, USA). Only completed responses were considered for analysis, and the normality of data was tested as appropriate using the Shapiro–Wilk test. A descriptive statistics method was used to calculate the percentage of responses for each question. Charts and tables were plotted in Microsoft Office Excel 365 according to data availability. Likert scales were analyzed using ordinal logistic regression, with stratification of years of practice, mode of practice, and others as independent factors to model the responses. Dichotomous survey questions were analyzed using binomial logistic regression (Supplementary Table S1). The significance level for all statistical tests was set at *p* < 0.05.

## 3. Results

Of the 462 responses obtained, 393 (85%) were valid. Invalid data excluded were 55 (12%) respondents with duplicate responses, 8 (1.7%) were undergraduate/diploma students, and 6 (1.3%) of the responses had missing items. Among the included responses, 49.4% (*n* = 194) were male, and 50.6% (*n* = 199) were female, with distribution spread across India (north 18%, south 50%, east 10%, and west 22%). Though respondents had a wide range of clinical experience, half of the participating optometrists were within their first five years (50.9%) of clinical practice.

Among the 393 respondents, 171 (43.6%) were undergraduate optometrists, 144 (36.6%) were postgraduate optometrists, 57 (14.5%) had diploma in optometry, and 20 (5.2%) were Ph.D. scholars/completed Ph.D. The majority of the respondents practiced at hospitals (40.4%, *n* = 159), with most of the optometrists (40.2%, *n* = 158) managing a maximum of five young children (<16 years of age) every week. The detailed characteristics of the respondents are listed in Table 2.

### 3.1. Knowledge of Childhood Myopia and Its Complications

Almost 90% of respondents indicated having a clinical/research interest in managing childhood myopia. The majority (45.3%) responded −0.50 D as the minimum correction in spherical equivalent prescribed for myopic patients. Most of the optometrists’ responses were retinal breaks (67.4%), followed by rhegmatogenous retinal detachment (45.3%) and cataracts (44.8%) as ocular pathologies associated with high myopia > −6 D (Table 3). Identification of retinal break was statistically significant among experienced respondents (≥ten years) (odds ratio (OR) 2.23, 95% confidence interval (CI): 1.53–3.25, *p* < 0.001), whereas it was not the case among respondents with interest in myopia management (OR 1.57, 95% CI: 0.60–4.05, *p* = 0.35). When stratified based on the primary place of practice, the odds of knowing retinal tear were equal within all practice patterns (academic institution (OR 2.14, 95% CI: 1.49–3.06, *p* < 0.001), corporate practice (OR 2.14, 95% CI: 1.53–2.99, *p* < 0.001), hospital (OR 1.79, 95% CI: 1.17–2.74, *p* = 0.007), independent practice (OR 2.11, 95% CI: 1.48–3.01, *p* < 0.001), and optometrists’ pursuing higher education (OR 1.98, 95% CI: 1.43–2.75, *p* < 0.001) (see details in Supplementary Table S1).

### 3.2. Clinical Workup and Diagnosis of Childhood Myopia

**Figure 1:**
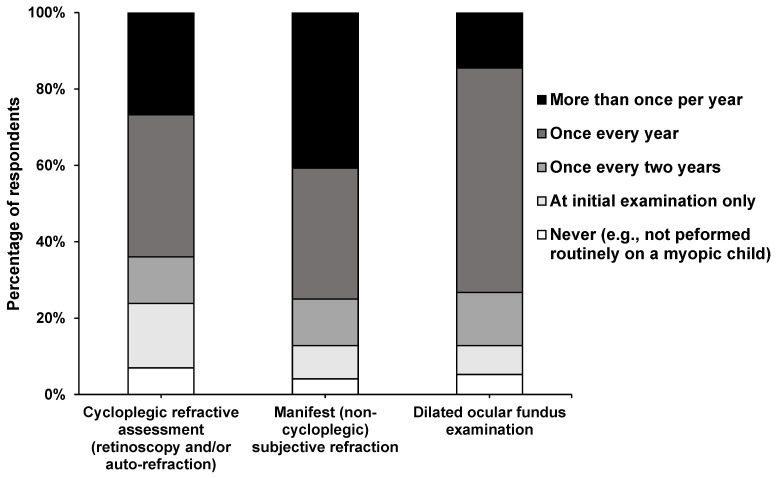
Percentage (%) of respondents who indicated performing each clinical procedure on a school-aged child with myopia at the nominated frequency.

Table 4 summarizes the responses to clinical procedures used to examine children with myopia for the first time. Over 75% of the respondents considered taking the family history, performing cycloplegic retinoscopy, dilated retinal examination, and cover test during the first visit. Respondents with myopia management interest and experience (≥10 years) were more likely to note the family history of myopia (OR 3.50, 95% CI: 1.15–10.6, *p* = 0.02 and OR 4.45, 95% CI: 2.81–7.07, *p* < 0.001) and perform cycloplegic refraction (OR 3.50, 95% CI: 1.15–10.60, *p* = 0.02 and OR 9.00, 95% CI: 4.96–16.30, *p* < 0.001) (see details in Supplementary Table S1). Ocular biometry is performed by 46.5% of practitioners in clinical practice. Notably, 7%, 4.1%, and 5.2% responded that they never performed cycloplegic, noncycloplegic, and dilated fundus examination procedures in the routine clinical practice on a myopic child (Figure 1).

### 3.3. Perception on Intervention

**Figure 2:**
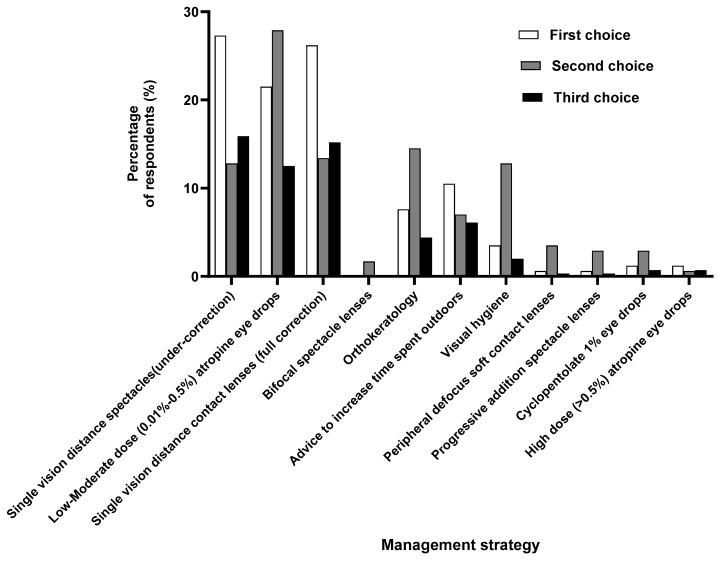
Perspective on the first three options for effective management other than single-vision spectacle (full-correction).

Table 5 presents the perspective on the most effective management options other than single-vision distance spectacles (full-correction) for myopia management. Experienced practitioners prioritized increasing time spent outdoors (OR 2.33, 95% CI: 1.58–3.45, *p* < 0.001), low–moderate-dose atropine (OR 2.24, 95% CI: 1.52–3.30, *p* < 0.001) and visual hygiene (OR 1.93, 95% CI: 1.32–2.81, *p* < 0.001) more than the inexperienced ones. Practitioners with an interest in myopia management were less likely to prescribe bifocal spectacle lenses with prism (OR 0.05, 95% CI: 0.01–0.44, *p* = 0.005), peripheral defocus soft contact lenses (OR 0.05, 95% CI: 0.01–0.44, *p =* 0.005), and progressive addition lenses (OR 0.20, 95% CI: 0.05–0.69, *p* = 0.01). Those working at hospitals were more likely to prescribe bifocal (OR 2.34, 95% CI: 1.30–4.23, *p* = 0.005) and visual hygiene (OR 1.97, 95% CI: 1.28–3.03, *p* = 0.002). The likelihood of advising increased time spent outdoors was significantly higher among academic, corporate, hospital, independent, and public health practices (OR 2.45–2.92, *p* < 0.001). Equally, low–moderate-dose atropine and visual hygiene were a preferred mode of advice among all the practices (OR 1.82–3.23, *p* < 0.001 and OR 1.93–2.04, *p* < 0.01, respectively) (see details in Supplementary Table S1). When asked about their perspective on the alternative to prescribing single-vision distance spectacles (full-correction), practitioners opted for single-vision (undercorrection) as their next choice, followed by single-vision contact lenses (full-correction), low–moderate-dose atropine (0.01–0.5%) eye drops, advice to increase the outdoor time, and Ortho-K (Figure 2).

### 3.4. Influential Factors on Management Approach

**Figure 3:**
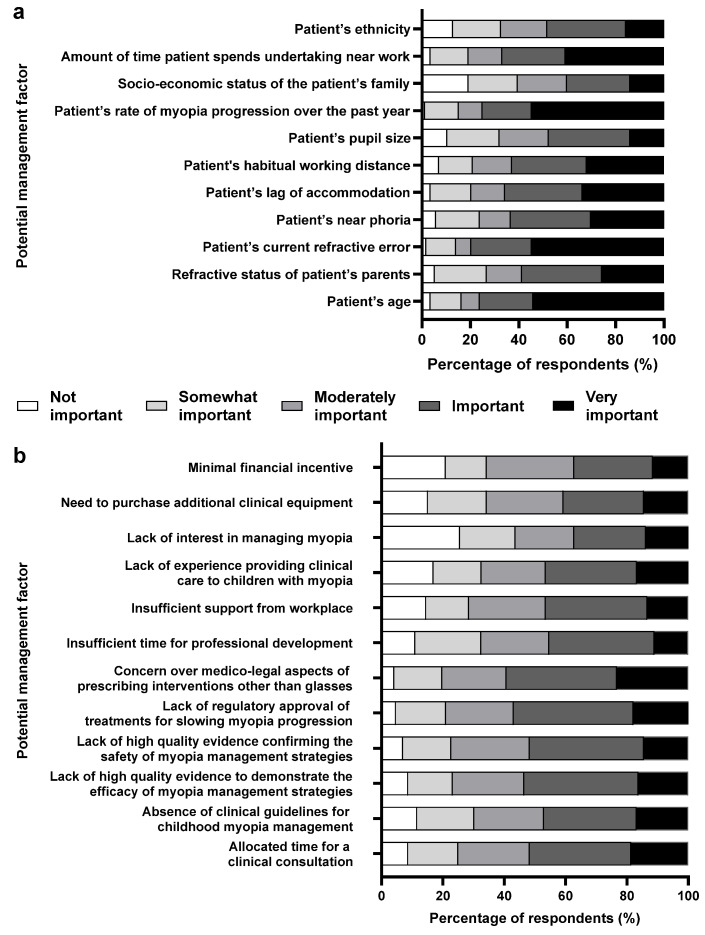
Percentage of respondents (%) who rated the relative importance of each: (**a**) potential factor when deciding upon the management approach for a child with myopia; (**b**) barrier limiting their ability to provide optimal clinical care to children with myopia.

Several clinical features of the myopic patient are deemed necessary by practitioners while managing myopes (Figure 3a). Over 50% of participants indicated that the age, current refractive error, and myopia progression over the past year are “very important”, followed by time spent in near work (40.7%).

Among the potential factors and barriers that influence optimal myopia care were concerns about the medicolegal implications of administering interventions, lack of regulatory approval of those treatments, scarcity of high-quality evidence backing up the effectiveness, inadequate consultation time, absence of clinical practice guidelines, and safety of myopia management techniques which were considered “very important” or “important” (Figure 3b). Experienced practitioners reported insufficient support at the workplace as a potential barrier (OR 1.88, 95% CI: 1.04–3.44, *p* = 0.03). Optometrists working at hospitals and academic institutions reported minimal financial incentives and lack of support from the workplace as significant barriers to myopia management (see details in Supplementary Table S1).

### 3.5. Management Approaches to Myopia in Children

**Figure 4:**
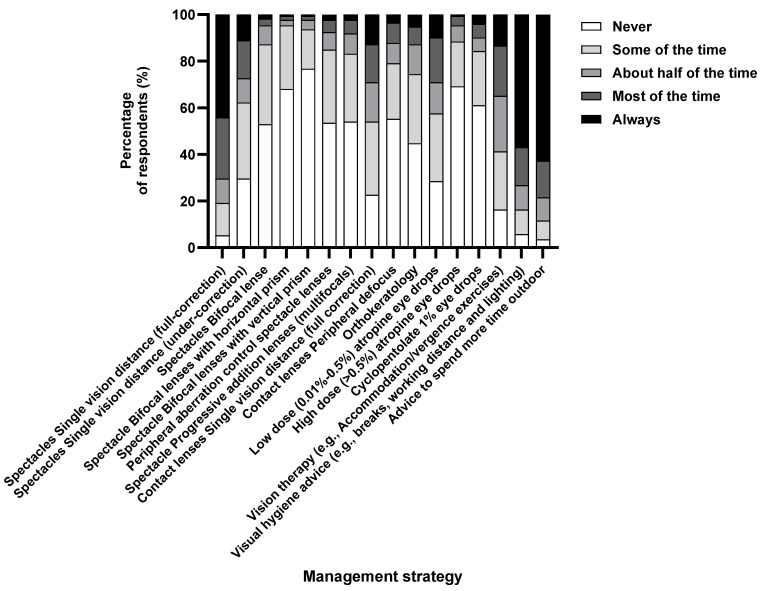
Percentage of respondents (%) who rated the frequency with which they prescribe each management strategy to children with myopia.

Single-vision distance (full-correction) spectacles were the most prescribed method of optical correction, with about 70.3% of respondents suggesting that they would “always” or “mostly” recommend this modality (Figure 4). Visual hygiene and increased outdoor time were equally prescribed as the management strategy to children with myopia.

Though the perception for low–moderate-dose atropine is effective (68.6%) in myopia management, it was prescribed relatively infrequently, with only 29.1% of practitioners reporting they would “always” or “mostly” recommend it. However, it was less likely that experienced optometrists would prescribe high-dose atropine (OR 0.40, 95% CI: 0.17–0.85, *p* = 0.02) and cyclopentolate (OR 0.49, 95% CI: 0.23–0.96, *p* = 0.04). In addition, the majority (>70%) of respondents indicated they would “mostly” or “always” advise on visual hygiene and increasing time spent outdoors. However, experienced optometrists were less likely to advise visual hygiene (OR 0.51, 95% CI: 0.27–0.95, *p* = 0.03).

Participants were asked to envision a clinical scenario for myopia management and specify when they would cease the intervention (patient age and prescription stability). Responses varied widely, with 16 years (14.5%) as the most common age criterion, and discontinuation was suggested if there was no progression in myopia for 12 months.

### 3.6. Source of Information

**Figure 5:**
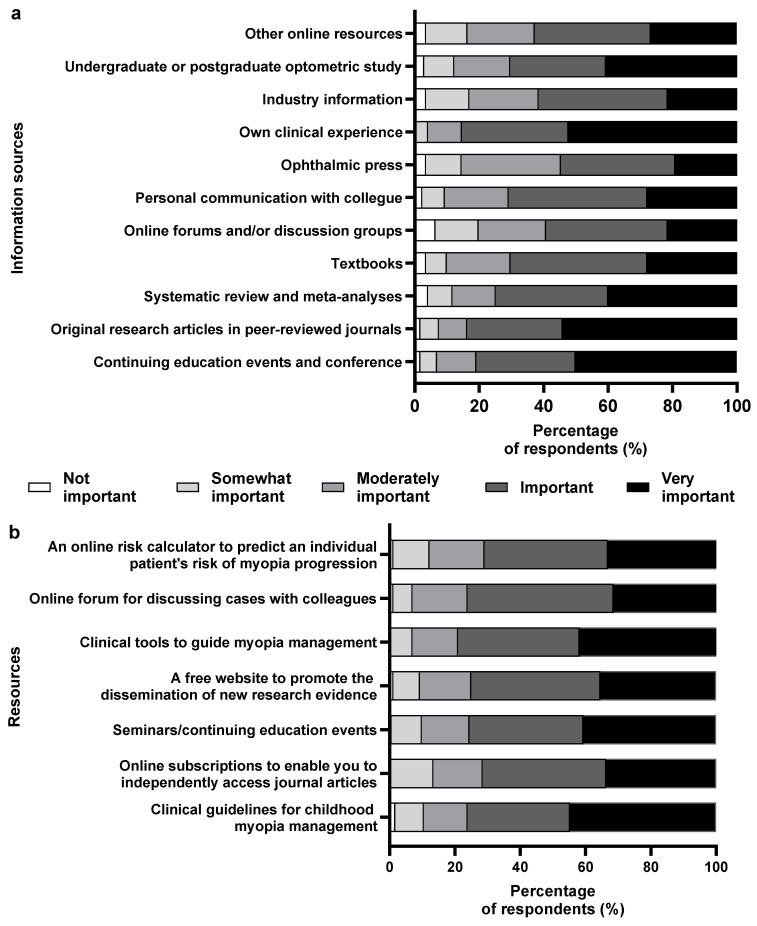
Percentage of respondents (%) who rated the relative importance of each: (**a**) information source in guiding their current approach to managing childhood myopia; (**b**) potential resources for supporting their future clinical management of children with myopia.

For most of the respondents (~85%), their own clinical experience was the source of information for clinical management was original peer-reviewed articles (~84%), followed by continuing medical education (CME) and conferences (~80%). The least opted sources were ophthalmic press (55%), online forums (~58%), industry information (~60%), and resources like artificial intelligence (AI) (~62%) (Figure 5a).

Among the resources that support the respondents’ future clinical management (Figure 5b), over 40% of the respondents mentioned clinical guidelines for childhood myopia management, seminars/continuing education events, and clinical tools to guide myopia management based upon the best available research evidence are very useful. Most (>70%) marked all listed items as “useful” or “very useful”. The results were similar when categorized based on interest, practice, and experience, without any significant difference.

### 3.7. Engagement with Caregivers

**Figure 6:**
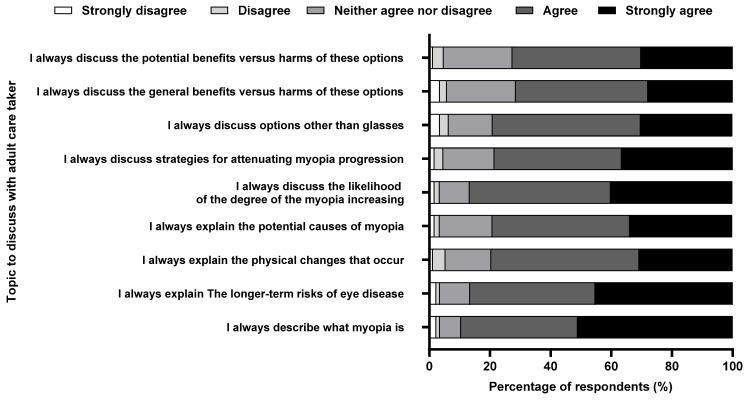
Percentage of respondents (%) who rated the relative importance of each potential topic to discuss with an adult caregiver of a child with myopia.

Over 40% emphasized discussing myopia’s nature, long-term eye disease risks, and potential myopia progression with adult caregivers. Over 70% found it essential to explain myopia, its physical changes, possible causes, increasing severity, and treatment benefits versus risks for a child. There were no significant differences based on experience or practice patterns, except for discussing treatment options. Regarding caregiver involvement in myopia management decisions, over 72% consider caregiver opinions or decide collaboratively after discussing management options (Figure 6).

## 4. Discussion

This survey provides an update and insight into the knowledge, perspective, and practice pattern of Indian optometrists towards childhood myopia management. This is the first study to analyze data from Indian optometrists with diverse practice patterns and clinical experience with equal gender representation (Table 2). The proportional distribution of the survey responses followed the distribution of optometry colleges across India [20]. The survey represented Indian optometry, encompassing practitioners from all states and union territories of India.

### 4.1. Complications of High Myopia

Overall, the knowledge level and awareness of Indian optometrists on the natural history of childhood myopia were comparable with their Australian and Spanish counterparts [14,15]. Though children and young teenagers with high myopia are at risk of developing visual impairment later in life due to the associated ocular pathologies [21], the survey shows that a significant proportion (32.6–92.4%) of respondents were unaware of the risk of ocular complications due to high myopia (Table 3). The awareness of the association of ocular pathologies with high myopia was underwhelming, with only 7.6–67.4% of Indian optometrists aware of the ocular complications, whereas it was 8.0–96.7% in Australia [14]. Our data showed increased awareness of ocular pathologies related to myopia compared to a previously described report (6.9–84.4%) [16], probably due to improved sampling and better survey penetration among various optometry groups (gender, years of experience, and practice) across India. Nevertheless, a lack of understanding about myopia complications, even among practitioners with an interest in myopia, implies that “interest” and “expertise” (knowledge) can be disconnected domains [22].

### 4.2. Risk Factors

The consideration of the child’s age, current refractive status, and rate of progression over the past year as “very important” or “important” by the majority (>70%) of the participants was consistent with the evidence available on the factors influencing myopia development [8,23]. However, to consider the ethnicity, parents’ refractive status, and time spent on near work as “moderate/somewhat important” was contrary and dismissive of the available evidence. The rate of myopia progression is faster among younger Asian children compared to other ethnicities [24], the risk of myopia is higher with one or more myopic parents [25], and clinical trials show that increasing time spent outdoors can reduce the development of myopia [8,26]. Over 83.7% of the respondents rightly indicated noting the family history of myopia, which, although was a relatively common practice, was lower than those reported in Australia and Spain (94.8–97.1%) [14,15].

### 4.3. Clinical Assessment

There is an improved acceptance of cycloplegic retinoscopy, autorefraction, and subjective refraction, with the majority of the participants (52.3–86.6%) performing it on initial examination (Table 4). The earlier study on Indian optometrists performed cycloplegic refraction at a lower level (34.1–63.6%) [16]. In comparison, cycloplegic refraction was infrequently practiced in Australia (11.7–17.2%) [14] and Spain (5.2–14.2%) [15,27]. Evidence suggests that cycloplegic refraction is the best practice to assess refractive error in children with active accommodation. Lack of cycloplegic refraction often leads to overestimation of myopic refraction by 0.63 D to 0.89 D [28]. Cycloplegic and noncycloplegic (manifest) subjective refraction was conducted yearly by 37.2% and 34.3% of the respondents, respectively, and more than once per year by 26.7% and 40.7%, respectively. Over 85% of the respondents were aware of the clinical relevance of cycloplegic refraction. Almost 35% of the respondents performed cycloplegic and noncycloplegic refraction yearly, and 40% performed noncycloplegic refraction more than once a year. Six per cent of respondents reported not performing cycloplegic refraction, and 4% did not perform manifest refraction. The understanding of the importance of pupil dilation and retinal examination, especially in high myopes, has also improved since the previous study (76% versus 53%) [16].

Ocular biometry in pediatric myopes is extremely important to estimate the progression and understand treatment efficacy [9]. Ocular axial length (AL) elongation has a strong association with axial myopia development [4] and is known to contribute to degenerative changes and ocular diseases related to pathological myopia [29]. Nevertheless, there is a gap between the knowledge and application among Indian optometrists, with more than half (53.5%) not measuring the AL. This aligns with reports from analogous surveys conducted across various nations globally. Axial length measurement was routinely performed by only a small proportion of optometrists in Australia (2.9%), Spain (13.5%), and Saudi Arabia (37%), primarily because optometric practice lacks accessibility to relatively expensive equipment like ocular biometers [14,15,30]. In addition, myopia control interventions are still a relatively new focus for optometrists. Limited clinical exposure, a lack of experience, and confidence in exhibiting clinical skills in myopia management might be additional barriers to performing ocular AL [31].

### 4.4. Management

Optometrists’ perception of the most effective myopia management option relative to regular spectacle lenses was time spent outdoors (73.8%), followed by atropine (68.6%), visual hygiene (66.3%), and orthokeratology (52.9%). In contrast, orthokeratology was perceived as the most effective myopia control treatment for children in Spain (51.3–55.5%) and Australia (93.3%), followed by pharmaceutical approaches like atropine, outdoor time, and soft contact lenses [14,15,27]. However, eye care practitioners in Singapore preferred atropine (53.1%) the most, followed by myopia control spectacles (30%) [32].

Furthermore, although optometrists perceived single-vision distance full-correction spectacles as an ineffective myopia control strategy, it was among the first three myopia management options most widely prescribed (~70%), along with visual hygiene (~70%) and advice to spend time outdoors (~76%) (Figure 4). This is consistent with reports from across the globe where optometrists most widely prescribe single-vision correction as the myopia control strategy [7,13,14,15,27]. The only exception is Singapore, where 80% of eye care practitioners actively prescribe newer myopia control strategies, with almost none prescribing single-vision spectacles [32]. Even though optical interventions such as undercorrection with single-vision lenses and progressive lenses for myopia control are not evidence-based [9] and might increase the rate of myopia progression [33], a proportion (27.9–36.6%) of respondents choose it secondary to single-vision full-distance correction spectacles. Most optometrists globally never use undercorrection (83.1%) [7]. However, a recent survey in Africa found that 52% of practitioners use undercorrection sometimes [13], which is alarming.

The survey reveals that Indian optometrists’ awareness of the efficacy [34] of low–moderate-dose atropine (68.6% versus 49%) and orthokeratology (53% versus 33%) has improved over the years [16] but still differs from practicing them. Most of the respondents appropriately advised increasing outdoor time for their myopia management option [35]. Despite the knowledge of the effectiveness of visual hygiene among experienced practitioners, there was a reduced likelihood of advising visual hygiene (i.e., taking visual breaks, having good lighting, and maintaining working distance). This reflects the difference between the level of understanding and practice behavior/clinical application. This dissociation between “knowledge” and “practice” may indicate a lack of confidence in visual hygiene and the need for recommended prescribing guidelines and knowledge base updation among relatively older practitioners [36].

Most respondents reported progressive lenses and peripheral defocus contact lenses to be efficient in myopia management. Although peripheral defocus contact lenses have demonstrated clinical efficacy in reducing myopia progression, progressive lenses demonstrated a subclinical difference to single-vision lenses and are, thus, not useful in clinical practice [28,37]. The primary concerns cited by the participating optometrists were possible medicolegal issues of prescribing newer interventions and lack of approval from regulatory bodies regarding the newer strategies, followed by the absence of high-quality evidence supporting the safety and efficacy of those myopia management strategies. Other barriers resulting in a deviation from the best available patient care included a need for more chair time for patients and the absence of a standard practice pattern or clinical guideline for managing myopia progression. This is consistent with the global report, where the perception of myopia control options as not effective (10% in Asia), unpredictable (>10% in Africa and Asia), requiring additional chair time (~10% in Asia and Europe), safety concerns (>20% in Africa and Asia), and cost to the patients (Africa 43.2%, Asia 33.2%, Europe 25.7%, Australasia 12.9%) were the main barriers [7].

### 4.5. Cessation

Responses considerably varied on the clinical scenario of treatment cessation criteria following myopia control; 16 years was the most common perceived age threshold with no myopia progression for 12 months. Australian optometrists seem to contradict the cessation criteria with most reporting 21 or 25 years of age, with no myopic progression for 24 months as the criteria to cease active intervention [14]. Myopia typically starts and progresses during childhood and adolescence (5–16 years), making this age group pivotal for understanding early detection and management strategies [8]. However, it should be recognized that myopia can still develop and progress in adolescents and young adults (>16 years), especially in those with high near-work demands [38]. Recent evidence suggests that stability of myopic refraction should be checked before discontinuation, which includes frequent follow-ups every six months to determine the chances of rebound effect and stability [39,40].

### 4.6. Source of Information

Similar to the Indian context, Australian optometrists ranked CME (>80%) and systematic reviews and meta-analyses (>75%) as their primary sources of clinical information [14]. Optometrists prefer preappraised and synthesized evidence over independently extracting and appraising the literature. This may indicate a need for more training, confidence, and time constraints in appraising the vastly available research evidence among participants [14,39]. Participants’ preference for resource materials corroborates with the previously available literature [14,41,42]. Notably, despite the extent of its popularity and widespread availability over smartphones, AI chatbots like ChatGPT (online resources) were not named as the common source of scientific information [43]. This was probably because these large language models are relatively new and have yet to gain trust among the optometric community, with possible awareness of their limitations in providing accurate or updated scientific evidence [44].

### 4.7. Caregiver

Most optometrists (40–72%) involve the adult caregiver and consider them essential in their child’s myopia management plan. It is in comparison to that in Australia, where 50–82% of practitioners reported informing adult caregivers about myopia and engaging them by discussing the management options [14]. The information–motivation–behavior theory indicates that when provided with high-quality information, individuals can experience shifts in their attitudes, fostering motivation for behavioural change and potentially leading to tangible behavioural changes [45]. The likelihood of these changes is influenced by the accessibility and stability of one’s attitude. Hence, a positive parental attitude, cultivated through informed decision making, can foster healthy habits and practices concerning their and their children’s vision. This proactive approach may contribute to a decrease in the prevalence and occurrence of childhood myopia [46,47]. A Singaporean study concluded that eye care practitioners strongly influence parents’ uptake of myopia control interventions, with 78.8% of parents following the recommendation [32]. Conversely, poor uptake of the recommended myopia control strategies by the parents or caregivers (24.4%) for single-vision lenses may be another challenge in using myopia control [32].

While we acknowledge the limitation that self-reporting may not reflect actual practice, the strength of this study is the relatively large sample size and responses from most states and union territories, thus representing the diverse practice pattern in India [17]. Compared to the previous report, which was confined regionally to a few Northern Indian states [16], the current study utilized a diverse network of state and national optometric associations, trusts, and social media platforms, with follow-up reminders sent every two weeks. This ultimately achieved deeper penetration by reaching a varied group of optometrists with wide-ranging years and modes of optometric practice across India. The questionnaire’s content was validated [14] and was pilot-tested to be contextually relevant, appropriate, and unambiguous. In contrast, the earlier study [16] was limited by insufficient methodological details of adopting the original Australian questionnaire into Indian optometric practice. The enhanced sampling and inclusion of optometrists from all strata of clinical experience and background of practice in this study might have elucidated a relatively higher proportion of respondents with better knowledge, attitude, and practice patterns. Nevertheless, the electronic questionnaire may have constrained responses to optometrists with digital literacy, and the voluntary nature of the survey likely led to a higher participation of optometrists engaged in myopia management, potentially introducing bias to the results. In addition, optometrists’ respective roles and competencies within each surveyed country we discussed were different, and their responses varied accordingly [48]. We limited this investigation to children aged 5–16 years, as this is the commonly reported age group when myopia develops and progresses [8]. Understanding how optometrists diagnose and manage myopia in this vulnerable age group provides deeper insights into early intervention strategies. Moreover, limiting the age range to 16 years or younger helps to focus the study on a specific developmental stage. It also aligns with the existing literature and guidelines on childhood myopia management [38]. While this study focused on children up to 16, it is essential to recognize that myopia management practices are also relevant for older adolescents and young adults. It is well established that myopia can still develop and progress in young adults, particularly in university student populations and in occupations with high near-work demands [38]. Future research may explore clinical practice behaviors in these older age groups to comprehensively understand myopia management across different developmental stages.

Through this study, we gained valuable insights into the current clinical practice behavior related to myopia diagnosis and management in children aged 16 years or younger and potential barriers in prescribing newer myopia management options among Indian optometrists. This understanding informs strategies for early intervention and better management of myopia to prevent possible long-term complications. While our knowledge informs common strategies for early intervention and enhanced myopia management to mitigate long-term complications, optometrists are apparently unable to fully embrace emerging myopia control strategies as a fundamental aspect of their clinical eye care service. Factors such as education, training, patient financial constraints, time limitations, and the limited availability of myopia control therapies emerge as prominent barriers to widespread adoption [49]. Despite abundant research evidence on myopia management, a lack of confidence to appraise a research article (insufficient time for professional development) and inadequate time for research in clinical practice (insufficient support from the workplace) are the other potential barriers to not being updated with the best clinical practice. Lifelong learning and continuing professional development (CPD) are linked to enhancing knowledge, skills, and performance to deliver optimum healthcare [50,51]. However, very little is known about the allocated or protected time to attend training for practicing optometrists. Workload and lack of support from the management are barriers to finding time for research and CPD [31]. Additionally, there are no published standard clinical practice guidelines for myopia control from optometry boards, councils, and associations in India, indicating the need to improve the prescribing guidelines to provide evidence-based care. This study underscores the pressing need for alignment between optometric training and the growing public health imperative for effective myopia control. Finally, although the questionnaire did not include the cost of myopia control, studies in Africa and Spain have reported increased financial costs to patients (e.g., a parent’s limited budget) as a significant reason for not prescribing or having poor acceptance of myopia control strategies [13,15,27], which might be the same in the Indian context.

## 5. Conclusions

This study provides valuable insight into the current knowledge, attitude, and practice of Indian optometrists in managing childhood myopia. The findings highlight the unawareness of high myopia complications, inconsistency in identifying risk factors, and the scope for essential clinical assessment to be improved. Although myopia management perspectives have improved, the uptake of those techniques in clinical practice still needs to accelerate. While optometrists are aware of emerging myopia interventions, single-vision distance correction remains the primary choice of vision correction. The published literature is the primary source of information for Indian optometrists, not AI chatbots. Barriers to optimal myopia care include medicolegal issues, lack of evidence-based clinical guidelines, and limited consultation time. Health regulatory bodies and the industry must collaborate and establish evidence-based approval of myopia management options. Ensuring access to clinical guidelines and CPD is required to remove the barriers to optimal care for myopia [7]. Myopia rates have risen since COVID-19 due to reduced outdoor activity and increased screen time [35]. These barriers must be overcome to advance global eye health and put available evidence into clinical practice.

## Figures and Tables

**Table 1:** Summary of the survey methodology.

S.no	Description	Survey Report
1	Survey development	A prevalidated questionnaire was revised after a focus group discussion among four optometrists to finalize the questionnaire’s items.
2	Number and nature of items in the questionnaire	A 26-item questionnaire with a mix of open- and closed-ended questions.
3	Reliability	Not determined.
4	Mode of survey	Internet-based.
5	Survey period	27 March 2020 to 27 September 2022.
6	Sample frame	Open survey: Open for all optometrists across India.
7	Target population	Optometrists practicing in India.
8	Recruitment process	Open invitations over social media, namely, Facebook, WhatsApp, E-Newsletter, and Telegram, in a target group with reminder messages every 2 weeks.
9	Participation	Voluntary participation.
10	Survey administration	Sequential questions administered using Google Forms.
11	Informed consent	E-consent.
12	Incentives	None.
13	Randomization of items or questionnaires	No randomization of items or questions.
14	Use of adoptive questions	Yes.
15	Number of screens	5.
16	Review step	Review with a back button, no alteration was possible after submission.
17	Preventing multiple entries from a single respondent, e.g., cookies used	Limited to one response per email and one item in the questions asked for their previous participation in the survey.
18	Data capturing	Automatic conversion into a spreadsheet.
19	Data analysis	Proportions of each response were calculated, and the odds were determined (*p* value of <0.05 was considered statistically significant).
20	Software used for statistical analysis	GraphPad Prism (version 6.04 for Windows, GraphPad Software, La Jolla, CA, USA and IBM SPSS Statistics for Windows, Version 28.0, IBM Corp.

**Table 2:** Demographics of the survey responders.

Characteristic	Respondents (*n* = 393)	*p*-Value *
Sex (male/female): *n* (%)	194/199 (49.4%/50.6%)	-
Optometric practice experience: *n* (%)		
0–5 years	200 (50.9%)	<0.001
5–10 years	62 (15.8%)	
10–15 years	40 (10.2%)	
15–20 years	44 (11.2%)	
>20 years	47 (12.0%)	
Primary place of optometric practice: *n* (%)		
Hospital	159 (40.4%)	<0.001
Academic Institution	63 (16.0%)	
Independent (private) practice	82 (20.9%)	
Corporate practice	52 (13.2%)	
Optometrist pursuing post-graduation	37 (9.5%)	
Possess a clinical or research interest in managing childhood myopia (yes/no): *n* (%)	352/41 (89.6%/10.4%)	
Number of myopic patients under 16 years of age provided care to in a typical week: *n* (%)		
0–5 6–10 11–15 16–20 21–25 26–30 >30	158 (40.2%) 114 (29.0%) 46 (11.7%) 16 (4.1%) 18 (4.6%) 9 (2.3%) 32 (8.1%)	<0.001

* Pearson chi-square test.

**Table 3:** Perspective on the associated ocular pathologies with high myopia (−6.00 D or greater).

Ocular Conditions	Percentage of Respondents (%)
Retinal breaks	67.4
Rhegmatogenous retinal detachment	45.3
Cataract	44.8
Exudative retinal detachment	30.8
Primary open angle glaucoma	29.7
Primary angle closure glaucoma	18.6
Age-related macular degeneration	16.9
Diabetic retinopathy	7.6

**Table 4:** Percentage of respondents who indicated performing each clinical procedure routinely on all school-aged children (5–16 years) with myopia on initial presentation.

Clinical Procedures	Percentage of Respondents (%)
Cycloplegic retinoscopy	86.6
Note patient family history of myopia	83.7
Dilated retinal fundus examination	76.7
Cover test (distance and near phoria)	75.0
Cycloplegic subjective refraction	70.9
Noncycloplegic retinoscopy	62.8
Noncycloplegic subjective refraction	62.2
Cycloplegic autorefraction	52.3
Intraocular pressure	47.1
Axial length measurement	46.5
Dynamic retinoscopy (e.g., MEM, NOTT retinoscopy)	39.5
AC/A ratio	35.5
Noncycloplegic autorefraction and stereopsis	34.3
Measurement of pupil size	32.6
Corneal topography	21.5
Retinal fundus photography—posterior pole	14.5
Retinal fundus photography—periphery	11.6
Peripheral defocus	8.7
Others	11.2

MEM: monocular estimation method; AC/A: accommodative convergence over accommodation.

**Table 5:** Perspective on the most effective management options of myopia in children other than single-vision distance spectacle (full-correction).

Myopia Management Option	Percentage of Respondents (%)
Advice to increase time spent outdoors	73.8
Low- to moderate-dose (0.01–0.5%) atropine eye drops	68.6
Visual hygiene (e.g., taking regular breaks with prolonged near work	
Maintaining appropriate working distance and good lighting	66.3
Orthokeratology	52.9
Single-vision distance spectacles (undercorrection)	36.6
Progressive addition spectacle lenses (multifocal)	27.9
Peripheral defocus soft contact lenses (e.g., distance-center multifocal soft contact lenses)	26.7
Bifocal spectacle lenses	20.3
Cyclopentolate 1% eye drops	11.6
Bifocal spectacle lenses with prism	9.3
High-dose (>0.5%) atropine eye drops	5.8
Others	4.7

## Data Availability

The raw data supporting the conclusions of this article will be made available by the authors on request.

## Supplementary Materials

The following supporting information can be downloaded at: https://www.mdpi.com/article/10.3390/vision8020022/s1, Table S1: Predictive factors for optometrists involving childhood myopia management based on their primary place of practice.
