# Supplementary material for: An Insight into Knowledge, Perspective, and Practices of Indian Optometrists towards Childhood Myopia
Source: Vision (Basel). 2024 Apr 16;8(2):22. doi: 10.3390/vision8020022 (PMC11036249; doi:10.3390/vision8020022)
Supplement: Supplementary file 1 [file vision-08-00022-s001.zip › vision-2899727-supplementary.pdf]

Supplementary Materials: An insight into knowledge, perspective, and practices of Indian optometrists towards childhood myopia

Archana Naik, Siddharth K. Karthikeyan, Jivitha Jyothi Ramesh, Shwetha Bhaskar, Chinnappa A. Ganapathi, Sayantan Biswas

Supplementary table S1: Predictive factors for optometrists involving childhood myopia management based on their primary place of practice.

Categories	Hospital			Academic institution			Independent (private) practice			Corporate practice			Optometrist pursuing PG		
	OR	95% CI	p-value	OR	95% CI	p-value	OR	95% CI	p-value	OR	95% CI	p-value	OR	95% CI	p-value
**Routine Eye Examination**															
Cover test	2.83	1.78-4.51	**<0.001**	2.94	2.01-4.32	**<0.001**	2.68	1.85-3.90	**<0.001**	3.36	2.32-4.88	**<0.001**	3.13	2.18-4.49	**<0.001**
Cycloplegic retinoscopy	5.57	3.15-9.84	**<0.001**	5.57	3.5-8.87	**<0.001**	6.78	4.13-11.10	**<0.001**	7.26	4.50-11.70	**<0.001**	6.67	4.21-10.50	**<0.001**
Cycloplegic subjective refraction	2.17	1.40-3.37	**0.001**	2.63	1.81-3.82	**<0.001**	2.33	1.63-3.35	**<0.001**	2.57	1.81-3.64	**<0.001**	2.50	1.78-3.52	**<0.001**
Dilated retinal fundus examination	2.29	1.47-3.56	**<0.001**	2.94	2.01-4.32	**<0.001**	4.19	2.75-6.37	**<0.001**	3.62	2.47-5.29	**<0.001**	3.47	2.40-5.03	**<0.001**
Non cycloplegic retinoscopy	1.56	1.02-2.36	**0.039**	1.76	1.24-2.49	**0.001**	1.86	1.31-2.63	**<0.001**	1.62	1.17-2.23	**0.003**	1.64	1.19-2.25	**0.002**
Non cycloplegic subjective refraction	1.56	1.02-2.36	**0.039**	1.76	1.24-2.49	**0.001**	1.80	1.27-2.54	**0.001**	1.57	1.14-2.17	**0.006**	1.56	1.13-2.13	**0.006**
Note patient family history of myopia	9.22	4.64-18.30	**<0.001**	4.75	3.06-7.38	**<0.001**	4.83	3.11-7.50	**<0.001**	5.04	3.31-7.67	**<0.001**	5.19	3.41-7.90	**<0.001**
**Practitioners' engagement with the attender**															
Always discuss options other than glasses for managing the child's myopia	0.88	0.50-1.54	0.652	2.3	1.15-4.70	**0.020**	0.67	0.33-1.33	0.251	0.868	0.32-2.37	0.781	1.47	0.44-5.08	0.531
**Considering risk factors**															
Patient's age	0.54	0.30-0.95	**0.033**	3.27	1.46-8.14	**0.006**	1.06	0.51-2.27	0.868	1.12	0.43-3.16	0.818	0.85	0.29-2.69	0.768
Patient's ethnicity	0.56	0.32-0.95	**0.034**	2.13	1.08-4.25	**0.031**	1.80	0.87-3.76	0.114	1.01	0.42-2.43	0.990	1.57	0.52-4.76	0.420
**Interventions**															
Advice to increase time spent outdoors	2.83	1.78-4.51	**<0.001**	2.45	1.7-3.54	**<0.001**	2.78	1.91-4.05	**<0.001**	2.92	2.04-4.19	**<0.001**	2.66	1.88-3.76	**<0.001**
Low-moderate dose 0.01%-0.5% atropine eye drops	1.87	1.22-2.88	**0.004**	1.82	1.28-2.57	**0.001**	2.26	1.58-3.23	**<0.001**	2.41	1.71-3.40	**<0.001**	2.22	1.59-3.10	**<0.001**

Visual hygiene e.g., taking regular breaks with prolonged near work maintaining appropriate working distance and good lighting	1.97	1.28-3.03	**0.002**	1.94	1.36-2.75	**<0.001**	2.04	1.44-2.91	**<0.001**	1.80	1.30-2.50	**<0.001**	1.93	1.39-2.67	**<0.001**
**Source of education and information**															
Systematic review and meta-analyses, Cochrane reviews	1.04	0.60-1.81	0.886	2.62	1.27-5.59	**0.010**	0.53	0.26-1.08	0.079	0.809	0.32-2.09	0.655	0.65	0.19-2.30	0.491
Textbooks	1.39	0.80-2.43	0.238	0.47	0.24-0.92	**0.028**	0.87	0.43-1.73	0.681	1.80	0.68-4.96	0.242	0.45	0.14-1.37	0.158
**Frequencies of interventions**															
Single vision distance (under-correction)	1.52	0.88-2.62	0.132	0.44	0.22-0.87	**0.020**	0.54	0.28-1.05	0.070	1.42	0.53-3.73	0.474	0.72	0.23-2.20	0.564
Bifocal lenses	2.34	1.30-4.23	**0.005**	0.62	0.29-1.29	0.215	0.72	0.33-1.51	0.387	0.38	0.10-1.05	0.108	0.87	0.25-2.72	0.816
**Awareness about complication**															
Retinal Break	1.79	1.17-2.74	**0.008**	2.14	1.49-3.06	**<0.001**	2.11	1.48-3.01	**<0.001**	2.14	1.53-2.99	**<0.001**	1.98	1.43-2.75	**<0.001**
**Barriers for myopia management**															
Insufficient support from workplace	0.63	0.37-1.08	0.091	2.72	1.36-5.56	**0.005**	0.74	0.38-1.43	0.366	1.30	0.507-3.35	0.586	1.90	0.56-6.74	0.309
Minimal financial incentive	1.77	1.03-3.04	**0.038**	1.07	0.54-2.11	0.848	0.55	0.27-1.10	0.09	0.95	0.39-2.31	0.91	1.35	0.40-4.60	0.624

PG: Post graduation
